# Brain natriuretic peptide and cardiac troponin I for prediction of the prognosis in cancer patients with sepsis

**DOI:** 10.1186/s12871-021-01384-9

**Published:** 2021-05-24

**Authors:** Yong Yang, Jiahua Leng, Xiuyun Tian, Hongzhi Wang, Chunyi Hao

**Affiliations:** 1 Department of Critical Care Medicine, Peking University Cancer Hospital & Institute, Beijing, People’s Republic of China; 2 Department of Hepato-Pancreato-Biliary Surgery, Peking University Cancer Hospital & Institute, Beijing, People’s Republic of China; 3 Gastrointestinal Cancer Center, Peking University Cancer Hospital & Institute, Beijing, People’s Republic of China; 4 Key laboratory of Carcinogenesis and Translational Research, (Ministry of Education/Bejing), Beijing, China

**Keywords:** Brain natriuretic peptide, Cardiac troponin I, SOFA score, Fluid balance, Sepsis, Cancer patients

## Abstract

**Background:**

This article aimed to study the value of brain natriuretic peptide (BNP) and cardiac troponin I(cTnI) for predicting the prognosis in cancer patients with sepsis.

**Methods:**

A cohort of 233 cancer patients with sepsis admitted to our ICU from January 2017 to October 2020 was included in this retrospective study. The data of BNP and cTnI on the first day (d1) and the third day(d3) after entering ICU, blood lactate (Lac), procalcitonin (PCT), Leucocyte and Sequential Organ failure assessment (SOFA) scores within 24 hr of entering ICU, the incidence of septic shock, acute kidney injury(AKI), acute respiratory failure (ARF) or sepsis-induced myocardial dysfunction(SIMD) in ICU, fluid balance in 24 hr and 72 hr after entering ICU, time of mechanical ventilation(MV), length of stay, emergency surgery were collected. According to 28-day mortality, patients were divided into survival group (190 cases) and death group (43 cases). All the above variables were compared.

**Results:**

BNP was an independent predictor for the mortality in these patients (*P* < 0.05).While cTnI was not. BNP on d3 in 681.5 pg/ml predicted the mortality with a sensitivity of 91.5 % and a specificity of 88.7 %. All patients were divided into the new two groups following the cutoff value of BNP on d3(681.5pg/ml), and the survival curve showed a significant difference with Kaplan-Meier analysis (*P* < 0.05). BNP had statistical differences between four groups based on the comorbidities(septic shock, AKI, ARF or SIMD), but cTnI was not.

**Conclusions:**

BNP was a great predictor for the prognosis of cancer patients with sepsis, while cTnI was not.

**Supplementary Information:**

The online version contains supplementary material available at 10.1186/s12871-021-01384-9.

## Introduction

Sepsis is “a life-threatening condition that arises when the body’s response to infection injures its own tissues” [1]. Although more progress has been made in the treatment for sepsis, it is still one of the common causes of death in critically ill patients worldwide [2]. Early warning and active intervention for sepsis can significantly reduce mortality and improve prognosis [3]. Poor regulation of normal immune responses caused by sepsis can result in a variety of adverse reactions, including multi-system organ dysfunction in several cases [4]. Sepsis induced myocardial dysfunction(SIMD) is common, with an incidence of about 40 %, which usually indicates a significant poor prognosis in sepsis [2–5].The application of BNP and cTnI in congestive heart failure and acute coronary syndromes has been extensively recognized and accepted [6, 7]. The two cardiac biomarkers for predicting the prognosis of septic patients have also become a hot spot in domestic and foreign research [7–9], but the value of them for predicting the prognosis of sepsis is still controversial.

This retrospective study was designed to clarify the differences of BNP and cTnI for predicting the prognosis of cancer patients with sepsis.

## Methods

### Participants

The study was carried out in accordance with the Declaration of Helsinki and approved by the Ethics Committee of Peking University Cancer Hospital & Institute. Clinical data on 233 cancer patients with sepsis admitted to ICU from January 2017 to October 2020 who met the inclusion criteria were collected retrospectively(315 were screened, and 82 were excluded according to the exclusion criteria).

Inclusion criteria: Patient data were collected according to the 2016 European definition of sepsis and septic shock [1].Patients with sepsis were treated by active cluster treatment according to the guidelines of Surviving sepsis campaign(SSC) [3].

Exclusion criteria: life expectancy is less than 24 hr, acute coronary syndrome, chronic heart disease (such as severe hypertension, heart valve disease or arrhythmia, etc.), chronic liver and kidney insufficiency, cardiogenic or hemorrhagic shock.

Both clinical and biological data were gathered in the following period after entering ICU.

Clinical data: age, gender, Infection category, comorbidities including septic shock, acute kidney injury(AKI), acute respiratory failure (ARF) and sepsis-induced myocardial dysfunction (SIMD) after entering ICU, time of MV, length of stay in ICU ,24 hr and 72 hr fluid balance in ICU, and emergency surgery conditions.

Biological data: lactate, leucocyte and PCT obtained from the blood gas, blood routine and procalcitonin test when patients entered ICU. BNP and cTnI on the first day(d1) and the third day (d3) after entering ICU(The normal value was less than or equal to 100 pg/ml for BNP, less than or equal to 0.05 ng/mL for cTnI).

SOFA scores were recorded to assess the severity of all the patients’s condition within the first 24 hr of admission to the ICU.

### Interpretation for some definitions

Sepsis is meant by the loss of control of the body’s inflammatory response to infection leading to life-threatening organ dysfunction. Organ dysfunction is defined as an acute increase in the Sequential Organ Failure Assessment score (SOFA score ≥ 2 points) secondary to infection.

Septic shock is defined as refractory hypotension (patients still need vasopressor drugs to maintain mean arterial pressure ≥ 65mmHg after adequate fluid resuscitation) and blood lactate ≥ 2mmol/l. Patients with sepsis were treated with active cluster treatment according to the sepsis treatment guidelines of Surviving sepsis campaign (SSC) [3].

AKI is meant by any of the following: Increase in serum creatinine(SCr)by 0.3 mg/dL(26.5µmol/L) within 48 h. Increase in SCr to 1.5 times baseline, which is known or presumed to have occurred within the prior 7 days. Urine volume<0.5ml/kg/h for 6 h [10].

ARF is described as acute severe dysfunction of lung ventilation caused by various reasons. Arterial blood oxygen partial pressure (PaO2) is lower than 8 kPa (60mmHg). Or accompanied by carbon dioxide partial pressure (PaCO2) higher than 6.65 kPa (50mmHg).

SIMD is meant by left ventricular ejection fraction (LVEF) less than 50 %. The bedside echocardiogram results were collected within 72 hr of entering ICU [11].

### Statistical Analysis

Statistics, Version 26.0 (Armonk, NY: IBM Corp.) was used for statistical analysis. Data were analyzed as the mean ± standard deviation, number(percentage) or median (25th /75th percentile). Unpaired t test and Mann-Whitney U test were used to compare continuous variables and skewed distribution. A χ^2^ test was used to compare categorical variables. Significantly different variables in univariate analysis were included in COX regression analysis to select the independent risk factors of sepsis. Receiver operating characteristic curve (ROC curve) was used to identify the value of all the independent risk factors for the mortality of cancer patients with sepsis. The patients were divided into the new two groups according to the cut-off value obtained by Youden index in ROC curve, and the difference in survival curve was compared with the Kaplan-Meier method. *P* < 0.05 was considered statistically significant.

## Results

**Fig. 1:**
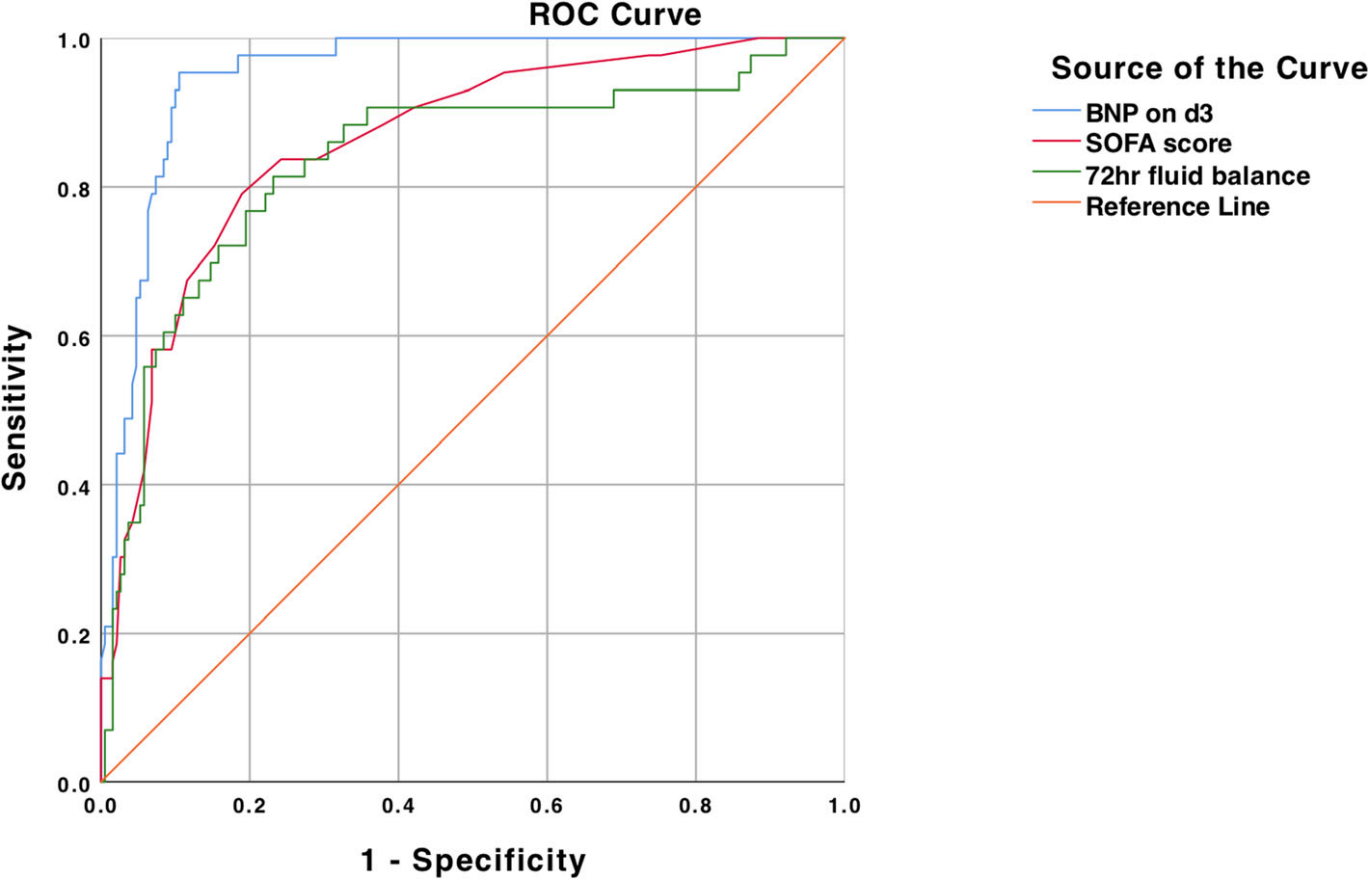
ROC curves of BNP on d3, SOFA score and 72 hr fluid balance for predicting the mortality of cancer patients with sepsis. The AUC of BNP on d3 was significantly larger than others (*P<*0.05)

1. According to the 28-day mortality, all cancer patients with sepsis were divided into the survival group and the death group. The baseline data for the two groups were as following (Table 1). The incidence of septic shock, AKI and ARF, the time of MV, 72 hr fluid balance, lactate, BNP and cTnI on d1 and d3, SOFA score in the survival group were significantly different from those in the death group by univariate analysis (*P* < 0.05).
2. The variables with significant differences in Table 1 were put into the Cox regression analysis. It can be seen that BNP on d3, SOFA score, and 72 hr fluid balance were independent risk factors for mortality of patients (Table 2).
3. The ROC curve was used to evaluate the predicting ability of the independent risk factors including BNP on d3, SOFA score, and 72 hr fluid balance from Table 2. The area under the ROC curve was 0.91 ± 0.01 (*P <* 0.01) for BNP on d3, 0.86 ± 0.03(*P <* 0.01) for SOFA score, 0.84 ± 0.04 (*P <* 0.01) for 72 hr fluid balance (Fig. 1). BNP on d3 at 681.5 pg/mL predicted mortality with a sensitivity of 91 % and a specificity of 89 %, SOFA score at 7 predicted mortality with a sensitivity of 79 % and a specificity of 81 %, 72 hr fluid balance at 75.9ml/kg predicted mortality with a sensitivity of 81 % and a specificity 77 %. It can be seen that BNP on d3 had the largest area of ROC curve, and it also had the best sensitivity and specificity.

**Table 1:** Baseline data for the survival and death groups of cancer patients with sepsis

	Total (*n* = 233)	Survival (*n* = 190)	death (*n* = 43)	P
**Sex, male**	169(72.5 %)	139(73.2 %)	30(69.8 %)	0.65
**Age(year)**	63.7 ± 9.9	63.5 ± 9.9	64.9 ± 10.1	0.4
**Infection category**				
** Respiratory**	79(33.9 %)	66(34.7 %)	13(30.2 %)	0.21
**Gastrointestinal**	17(7.3 %)	11(7.9 %)	4(9.3 %)	0.32
**Abdominal cavity**	99(42.3 %)	80(42.1 %)	18(41.9 %)	0.54
**Thoracic cavity**	27(11.6 %)	23(12.1 %)	7(16.2 %)	0.14
**Catheter related blood stream infection**	3(1.3 %)	3(1.6 %)	0	0.13
**Genitourinary**	5(2.1 %)	5(2.1 %)	0	0.21
**Others**	3(1.3 %)	2(1.1 %)	1(2.3 %)	0.16
**Septic shock**	94(40.3 %)	60(31.6 %)	34(79.1 %)	0.001
**AKI**	40(17.2 %)	14(7.4 %)	26(60.5 %)	0.001
**ARF**	123(52.3 %)	88(46.3 %)	35(81.4 %)	0.001
**SIMD**^a^	42/126 (33.3 %)	30/98(30.6 %)	12/28(42.9)	0.26
**Total MV time(day)**	3.6 ± 5.9	2.7 ± 4.7	7.5 ± 8.7	0.001
**ICU stay time(day)**	7.8 ± 5.9	7.7 ± 5.3	8.6 ± 7.7	0.46
**Fluid-balance(ml/kg)**				
**24 hr**	49.4 ± 35.8	46.1 ± 33.1	53.9 ± 43.3	0.083
**72 hr**	63.1 ± 54.9	50.5 ± 45.8	118.5 ± 58.2	0.002
**Emergency surgery**	62(26.7 %)	51(26.8 %)	11(25.6 %)	0.26
**Lactate(mmol/l)**	2.9 ± 2.2	2.6 ± 1.6	4.6 ± 3.5	0.001
**Leucocyte (10^9/l)**	13.3 ± 8.3	13.2 ± 8.4	13.7 ± 8.3	0.73
**PCT (ng/ml)**	17.6 ± 45.2	15.7 ± 43.6	25.4 ± 51.5	0.21
**Cardiac biomarkers**				
**BNP (pg/ml)**				
**d1**	673.6 ± 786.6	618.1 ± 724.7	919.0 ± 989.6	0.01
**d3**	656.6 ± 912.4	370.2 ± 456.9	1922.1 ± 1284.1	0.000
**cTnI(ng/ml)**				
**d1**	0.04(0.02/0.17)	0.03(0.01/0.16)	0.08(0.03/0.23)	0.04
**d3**	0.03(0.01/0.12)	0.02(0.01/0.05)	0.21(0.11/1.11)	0.02
**SOFA Score**	5(4/10)	4(3/7)	9(4/12)	0.000

Data were expressed as mean ± standard deviation, number (percentage), or median (25th/75th percentile). *AKI* acute kidney injury; *ARF* acute respiratory failure; *SIMD* sepsis-induced myocardial dysfunction; *CRRT* continuous renal replacement therapy; *MV* mechanical ventilation; *ICU* intensive care unit; *PCT* Procalcitonin; *BNP* brain natriuretic peptide; *cTnI* cardiac troponin I; *24hr* In 24hr after entering ICU; *72 hr* In 72 h after entering ICU; *d1* the first day in ICU; *d3* the third day in ICU; *SOFA* Sequential Organ failure assessment. ^a^126 out of 233 patients underwent bedside echocardiogram

**Table 2:** Cox regression analysis for cancer patients with sepsis

Variables	B	Wald	*P*-value	OR	95 %CI
**BNP on d3**	0.003	23.609	0.000	1.003	1.002–1.005
**SOFA score**	0.128	12.133	0.000	1.136	1.057–1.221
**72 h Fluid balance**	0.012	4.514	0.034	1.012	1.001–1.023

*BNP* brain natriuretic peptide; *d3* the third day in ICU; *SOFA* Sequential Organ failure assessment; *72 hr* In 72 h after entering ICU

**Fig. 2:**
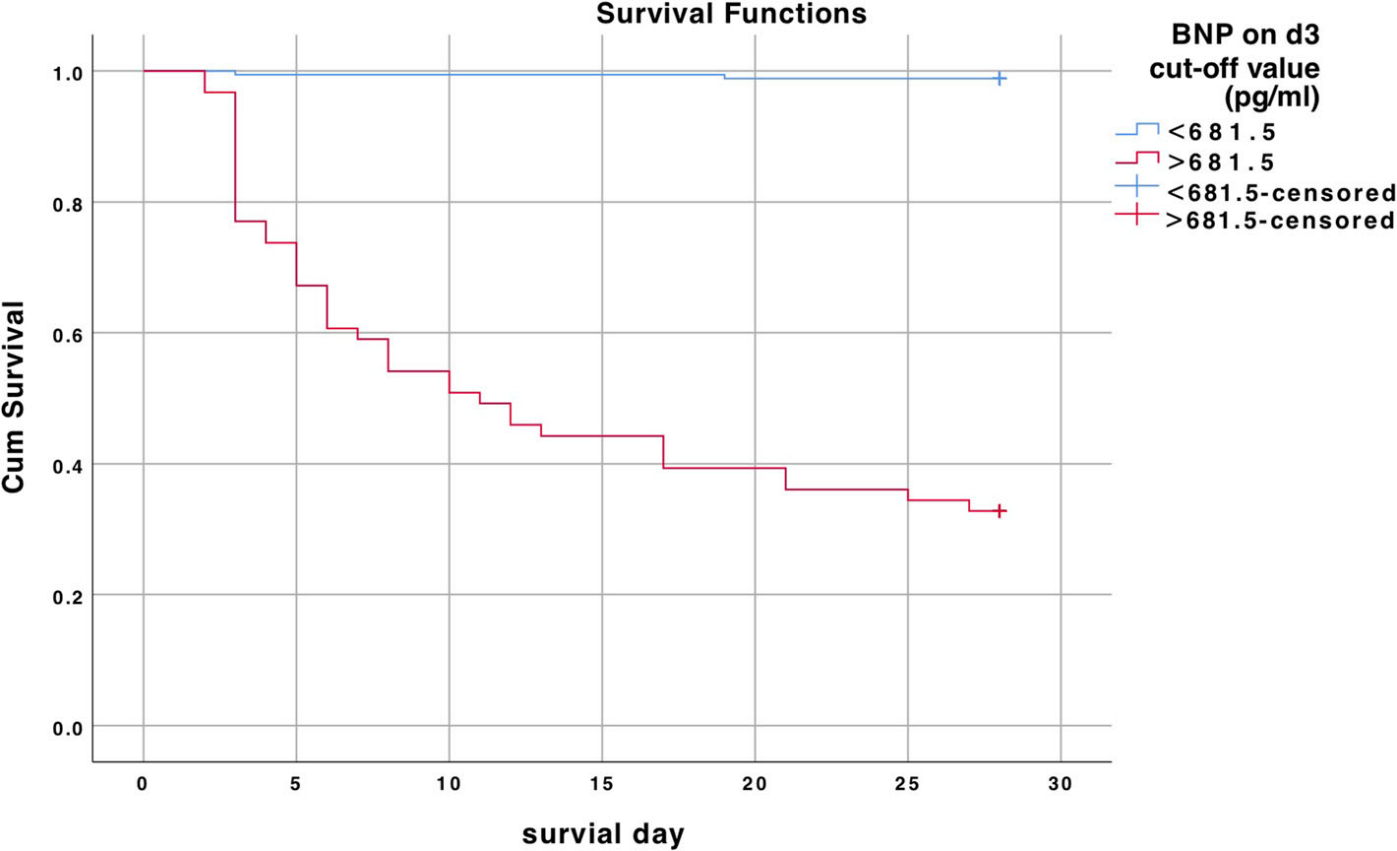
The new two groups (BNP on d3 < 681.5pg/ml and BNP on d3 > 681.5pg/ml) had a significant difference in the survival curve (*P* < 0.05)

**Fig. 3:**
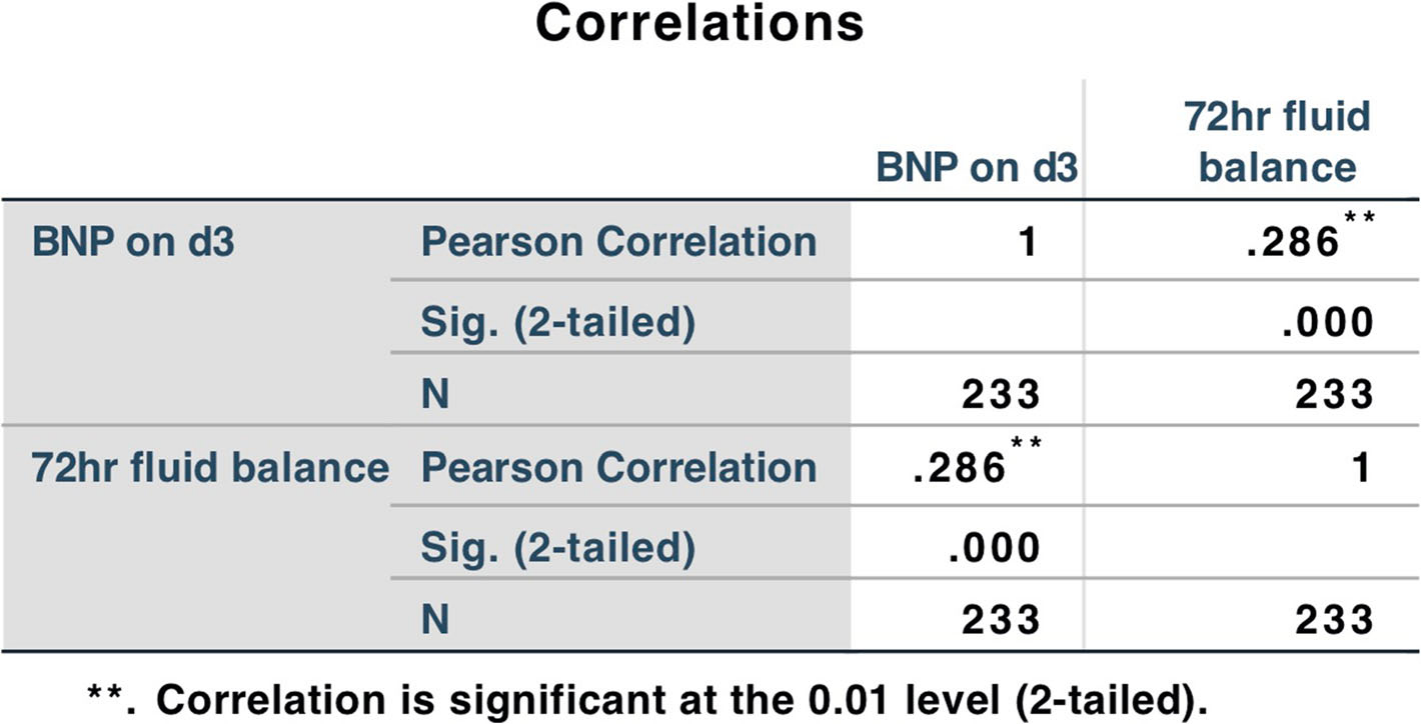
BNP on d3 and 72 hr fluid balance had a positive correlation (*P* < 0.05), but the correlation was extremely weak (*r* = 0.286)

4. According to the cut-off value of BNP on d3(681.5 pg/ml), all patients were divided into two groups (BNP on d3 < 681.5pg/ml or BNP on d3 > 681.5pg/ml), Kaplan-Meier analysis performed on the two groups of patients showed a significant difference in the survival curve (P < 0.05) which means that the greater the BNP on d3 above the cut-off value, the higher the 28-day mortality rate of the patients.(Fig. 2)..

5. There were also significant differences in the comorbidities (septic shock, AKI, ARF, SIMD) after entering ICU occurred in the two groups of patients (BNP on d3 < 681.5pg/ml or BNP on d3 > 681.5pg/ml) (*P* < 0.05), which indicated that the higher the BNP of the patients, the more likely to merge with shock, AKI, ARF or SIMD. (Table 3)
6. Differences of BNP and cTnI were compared among the four groups according to the comorbidities (septic shock, AKI, ARF, SIMD). It can be seen that the level of BNP increased significantly (*P* < 0.05) while cTnI was not in patients with comorbidities (Tables 4, 5, 6 and 7).
7. The correlation between BNP on d3 and 72 hr fluid balance was compared. Both of them had a positive correlation (*P* < 0.05), but the correlation was extremely weak (*r* = 0.286) (Fig. 3)..

**Table 3:** Comparison of comorbidities between the new groups of patients grouped by the cutoff value

BNP on d3 (pg/ml)	< 681.5(*n* = 172)	> 681.5(*n* = 61)	P
**Septic shock**			0.000
yes	53(30.8 %)	41(67.2 %)	
no	119(69.2 %)	20(32.8 %)	
**AKI**			0.001
yes	10(5.8 %)	30(49.2 %)	
no	162(94.2 %)	31(50.8 %)	
**ARF**			0.025
yes	83(48.3 %)	40(65.6 %)	
no	89(51.7 %)	21(34.4 %)	
**SIMD**^a^	< 681.5(n = 83)	> 681.5(n = 43)	0.043
yes	23/83(27.7 %)	19/43(44.2 %)	
no	60/83(72.3 %)	24/43(55.8 %)	

*BNP* brain natriuretic peptide; *d3* the third day in ICU; *AKI* acute kidney injury; *ARF* acute respiratory failure; *SIMD* sepsis-induced myocardial dysfunction; ^a^126 out of 233 patients underwent bedside echocardiogram

**Table 4:** Comparison of BNP and cTnI between the non-septic shock group and the septic shock group

	Non-septic shock (*n* = 139)	Septic shock (*n* = 94)	P
BNP on d1	482.3 ± 532.8	956.4 ± 993.3	0.008
BNP on d3	367.3 ± 402.4	1084.5 ± 1235.5	0.000
cTnI on d1	0.02(0.01/0.08)	0.08(0.02/0.30)	0.11
cTnI on d3	0.02(0.01/0.04)	0.06(0.02/0.32)	0.14

*BNP* brain natriuretic peptide; *cTnI* cardiac troponin I; *d1* the first day in ICU; *d3* the third day in ICU

**Table 5:** Comparison of BNP and cTnI between the non-AKI group and the AKI group

	Non-AKI (*n* = 193)	AKI (*n* = 40)	P
BNP on d1	583.1 ± 698.3	1110.0 ± 1021.9	0.011
BNP on d3	408.5 ± 469.7	1853.6 ± 1446.6	0.000
cTnI on d1	0.06(0.01/0.14)	0.09(0.03/0.14)	0.21
cTnI on d3	0.02(0.01/0.05)	0.08(0.04/0/19)	0.10

*BNP* brain natriuretic peptide; *cTnI* cardiac troponin I; *d1* the first day in ICU; *d3* the third day in ICU; *AKI* acute kidney injury

**Table 6:** Comparison of BNP and cTnI between the non-ARF group and the ARF group

	Non-ARF (*n* = 110)	ARF (*n* = 123)	P
BNP on d1	523.1 ± 718.5	858.8 ± 843.5	0.032
BNP on d3	475.1 ± 600.3	819.0 ± 1097.7	0.000
cTnI on d1	0.02(0.01/0.14)	0.05(0.02/0.20)	0.22
cTnI on d3	0.02(0.01/0.32)	0.03(0.01/0/21)	0.31

*BNP* brain natriuretic peptide; *cTnI* cardiac troponin I; *d1* the first day in ICU; *d3* the third day in ICU; *ARF* acute renal failure

**Table 7:** Comparison of BNP and cTnI between the non-SIMD group and the SIMD group

	Non-SIMD (*n* = 84)	SIMD (*n* = 42)	P
BNP on d1	753.0 ± 779.8	1191.2 ± 978.1	0.008
BNP on d3	748.7 ± 1004.6	1076.2 ± 1175.6	0.03
cTnI on d1	0.03(0.01/0.23)	0.04(0.02/0.31)	0.28
cTnI on d3	0.02(0.01/0.10)	0.06(0.04/0.29)	0.43

*BNP* brain natriuretic peptide; *cTnI* cardiac troponin I; *d1* the first day in ICU; *d3* the third day in ICU; *SIMD* sepsis-induced myocardial dysfunction

## Discussion

BNP is a definitive marker in patients with congestive heart failure. BNP is released into the blood when the ventricular wall tension increased[12]. The main mechanism of SIMD is that the heart’s variable myocardial contractility would increase the tension of the ventricular wall and cause ventricular dilatation, which leads to a significant increase in the secretion of BNP[13]. Patients with septic shock often have acute renal injury in the initial stage, and the metabolism of BNP produced in plasma is slowed down[14]. These two factors are the main reason why causing the increase of BNP in patients with sepsis. The main finding of this study was to understand that BNP is an independent risk factor for the prognosis of cancer patients with sepsis, especially the BNP on d3 after entering the ICU at 681.5pg/ml had high diagnostic power and great sensitivity and specificity for the mortality of these patients. The higher the BNP level larger than 681.5pg/ml, the higher the 28-day mortality rate, and the greater the possibility of the comorbidities (septic shock, AKI, ARF, SIMD) occurred. This result is consistent with some domestic and foreign studies [15, 16].

Differences of BNP and cTnI were compared among the four groups according to the comorbidities (septic shock, AKI, ARF, SIMD). It can be seen that the level of BNP increased significantly (*P* < 0.05) while cTnI was not in patients with comorbidities.

cTnI is the most sensitive and specific marker of myocardial injury. Cardiac hypoperfusion and the application of a large number of vasoactive drugs in patients with sepsis both may cause myocardial injury[17]. The degree of elevated cTnI was significantly related to the severity and mortality of patients with sepsis[18]. However, Some studies also had shown that cTnI has no obvious relationship with mortality of septic patients [19, 20]. This study found that cTnI was significantly different between the survival group and the death group (P < 0.05), but cTnI was not an independent risk factor predicting the mortality in patients with sepsis.

126 underwent random bedside echocardiography (the remaining were not available) among the 233 patients with sepsis in this study. A total of 42 cases developed SIMD, with an incidence rate of 33.3 % (30.6 % in the survival group and 40.9 % in the death group). There was no significant differences in the incidence of SIMD between the two groups (*P* = 0.26). And also there was no significant difference between the non-SIMD and SIMD groups for cTnI on d1 and d3. This conclusion was consistent with the results of RøSjø who found that the increase of cTnI in patients with sepsis only reflected the damage state of myocardial cells and cannot increase the mortality of sepsis or accurately predict the risk of SIMD [21]. Combining the above multiple studies, It can be observed that the value of cTnI for the prognosis of patients with sepsis is still controversial [17].

This study also found that the SOFA score and 72 hr fluid balance were independent risk factors for mortality in these patients. SOFA score is a reliable indicator to assess the severity of critical ill patients[22, 23]. Its predictive value for the mortality of patients with sepsis has been confirmed by a large number of studies and would not be discussed further here[24, 25]. 72 hr fluid balance was also one of the independent risk factors[26]. The area under the ROC curve was 0.84 ± 0.04 (*P <* 0.01) for 72 hr fluid balance. 72 hr fluid balance at 75.9ml/kg predicted mortality with a sensitivity of 81 % and a specificity 77 %. It can be seen that 72 hr fluid balance has good predictive value for the mortality of cancer patients with sepsis. In the early treatment of sepsis, in order to optimize organ perfusion, fluid shock therapy should be performed in time. But the continuous positive fluid balance in patients with sepsis in the following periods may indicate a poor prognosis. The European SOAP study in 2006 showed that the cumulative fluid balance within 72 hr is the strongest predictor of mortality of sepsis patients in the ICU, which means that fluid balance is the only changeable risk factor identified in their study [27]. Boyd reported a retrospective study of VASST, which also confirmed the relationship between the cumulative fluid balance after 4 days and the mortality of patients with sepsis [28].

The correlation analysis between BNP on d3 and 72 hr fluid balance showed that the two were positively correlated (P<0.05), but the correlation was extremely weak (*r* = 0.286). BNP didn’t seem to be a reliable marker of fluid status in septic patients. Similar studies had also shown that BNP was not closely associated with fluid volume and fluid responsiveness in patients with sepsis[29, 30].

### Limitations

This study referred to the latest definition of sepsis. Enrollment and grouping of sepsis patients had new standards, and the conclusions were different from previous studies. Dynamic observation data of BNP, cTnI and fluid balance increased the accuracy of the results. This study still had certain limitations. First, the enrolled patients had a short hospital stay in ICU, so most of the BNP and cTnI data were within 3 days of entering ICU. The dynamic observation data were relatively limited, which may influence the judgment of the results to a certain extent. Secondly, not all the patients had undergone bedside echocardiography, so the sample size was reduced. Because of the limited technology of bedside echocardiography, patients diagnosed with SIMD were actually based on left ventricular systolic dysfunction, which would lose some patients with left ventricular diastolic dysfunction or right heart dysfunction. The incidence of SIMD may be smaller. The difference of BNP and cTnI with SIMD, and mortality between SIMD and non-SIMD groups may be biased ultimately. In future, more sample size and more cardiac ultrasound parameters should be added. Prospective studies would be conducted to improve the rigor of the research.

## Conclusions

For cancer patients with sepsis, early warning and effective intervention to reduce mortality are still the difficulties in ICU. BNP is a great predictor for evaluating the prognosis of cancer patients with sepsis. While cTnI is still controversial. Early judgment on the prognosis of patients with sepsis still needs to look for more biomarkers to enhance their effectiveness.

## Supplementary Information

**Additional file 1:**

## Data Availability

The datasets used and analyzed during the current study are available from the corresponding author on reasonable request.

## Abbreviations

ICU: Intensive care unit
BNP: Brain natriuretic peptide
cTnI: Cardiac troponin I
d1: The first day
d3: The third day
Lac: Lactate
PCT: Procalcitonin
SOFA: Sequential Organ failure assessment
AKI: Acute kidney injury
ARF: Acute respiratory failure
SIMD: Sepsis-induced myocardial dysfunction
MV: Mechanical ventilation
72hr: 72 hour
24hr: 24 hour
SSC: Surviving sepsis campaign
SCr: Serum creatinine
PaO2: Arterial blood oxygen partial pressure
PaCO2: Carbon dioxide partial pressure
LVEF: Left ventricular ejection fraction
ROC: Receiver operating characteristic
