# Supplementary material for: Brain natriuretic peptide and cardiac troponin I for prediction of the prognosis in cancer patients with sepsis
Source: BMC Anesthesiol. 2021 May 24;21:159. doi: 10.1186/s12871-021-01384-9 (PMC8142482; doi:10.1186/s12871-021-01384-9)

Cox Regression Power Analysis

Numeric Results

Power	Sample Size (N)	Reg. Coef. (B)	S.D. of X1 (SD)	Event Rate (P)	R-Squared X1 vs Other X's (R2)	One-Sided Alpha	Beta
0.80242	104	1.5000	0.5000	0.1800	0.2500	0.02500	0.19758

References

Hsieh, F.Y. and Lavori, P.W. 2000. 'Sample-Size Calculations for the Cox Proportional Hazards Regression Model with Nonbinary Covariates', Controlled Clinical Trials, Volume 21, pages 552-560.
Schoenfeld, David A. 1983. 'Sample-Size Formula for the Proportional-Hazards Regression Model', Biometrics, Volume 39, pages 499-503.

Report Definitions

Power is the probability of rejecting a false null hypothesis. It should be close to one.
N is the size of the sample drawn from the population.
B is the size of the regression coefficent to be detected
SD is the standard deviation of X1.
P is the event rate.
R2 is the R-squared achieved when X1 is regressed on the other covariates.
Alpha is the probability of rejecting a true null hypothesis.
Beta is the probability of accepting a false null hypothesis.

Summary Statements

A Cox regression of the log hazard ratio on a covariate with a standard deviation of 0.5000 based on a sample of 104 observations achieves 80% power at a 0.02500 significance level to detect a regression coefficient equal to 1.5000. The sample size was adjusted since a multiple regression of the variable of interest on the other covariates in the Cox regression is expected to have an R-Squared of 0.2500. The sample size was adjusted for an anticipated event rate of 0.1800.

Cox Regression Power Analysis

Chart Section

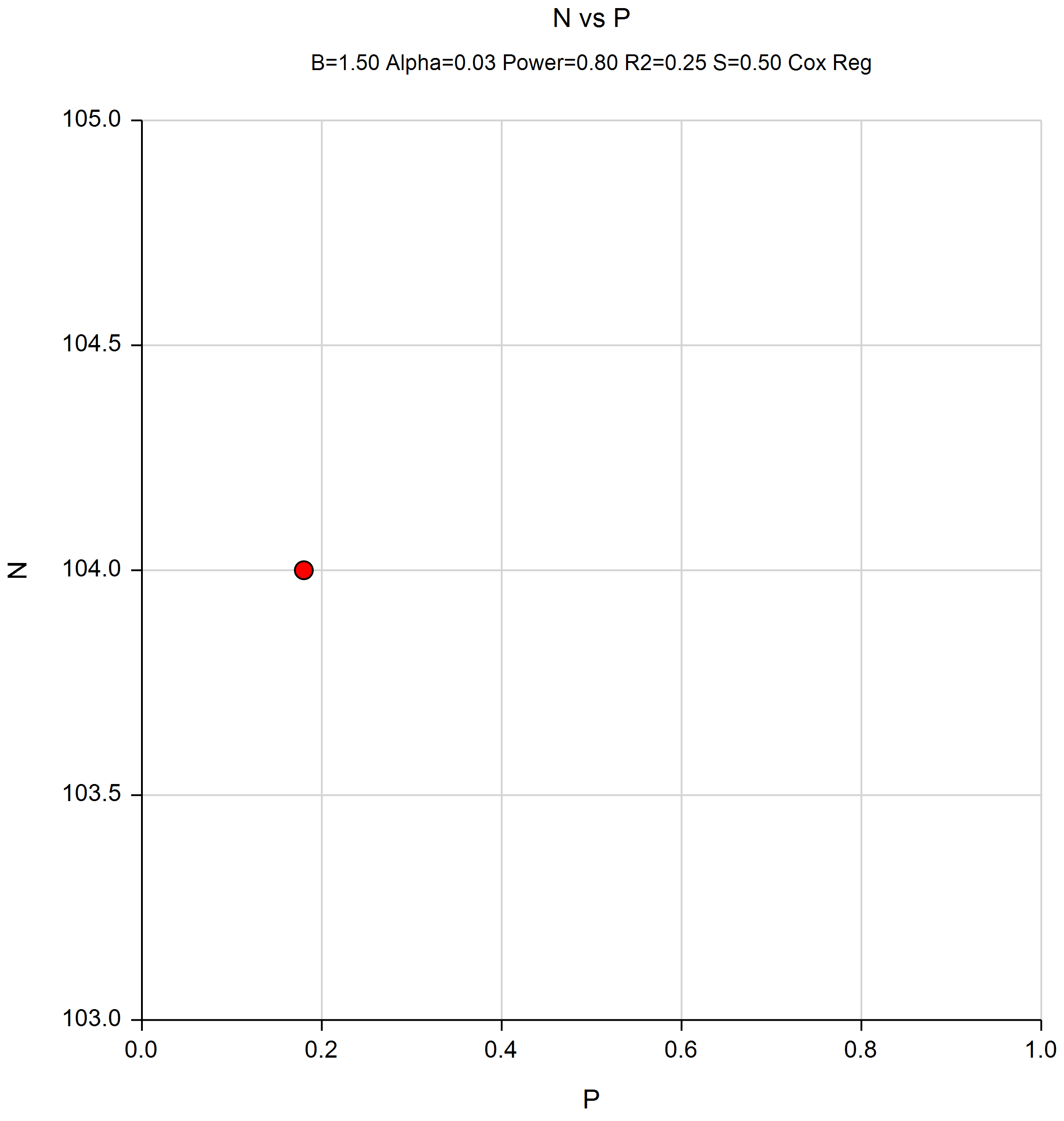

Supplement: Supplementary file 1 — Additional file 1: [file 12871_2021_1384_MOESM1_ESM.pdf]
